# Prevalence of burnout, depression, anxiety and stress in Australian midwives: a cross-sectional survey

**DOI:** 10.1186/s12884-016-1212-5

**Published:** 2017-01-09

**Authors:** D. K. Creedy, M. Sidebotham, J. Gamble, Julie Pallant, J. Fenwick

**Affiliations:** 1 Menzies Health Institute Queensland, School of Nursing & Midwifery, Griffith University, Logan Campus, University Drive, Meadowbrook, Qld 4131 Australia; 2 Gold Coast University Hospital, Southport, Australia

**Keywords:** Anxiety, Burnout, Copenhagen Burnout Inventory, Depression, Midwives, Midwife, Stress, Survey

## Abstract

**Background:**

The health and wellbeing of midwives are important considerations for workforce retention and quality care. The occurrence and relationships among mental health conditions such as burnout and depression have received little attention. We investigated the prevalence of burnout, depression, anxiety and stress in Australian midwives.

**Methods:**

An online survey was conducted in September 2014. Participants were recruited through the Australian College of Midwives and professional networks. The survey sought personal and professional details. Standard measures included the Copenhagen Burnout Inventory (CBI) (Personal, Work and Client subscales), and Depression, Anxiety, and Stress Scale (DASS). The sample was collapsed into two groups according to DASS clinical cut-offs (normal/mild versus moderate/severe/extreme). Effect size statistics were calculated and judged according to Cohen’s guidelines.

**Results:**

One thousand thirty-seven surveys were received. Respondents were predominantly female (98%), with an average age of 46.43 years, and 16.51 years of practice. Using a CBI subscale cut-off score of 50 and above (moderate and higher), 64.9% (*n* = 643) reported personal burnout; 43.8% (*n* = 428) reported work-related burnout; and 10.4% (*n* = 102) reported client-related burnout. All burnout subscales were significantly correlated with depression, anxiety and stress, particularly personal and work-related burnout with Spearman’s rho correlations ranging from .51 to .63 (*p* < .001). Around 20% of midwives reported moderate/ severe/ extreme levels of depression (17.3%); anxiety (20.4%), and stress (22.1%) symptoms. Mann-Whitney U tests revealed significant differences between groups with depression (*r* = .43), anxiety (*r* = .41) and stress (*r* = 48) having a medium size effect on burnout.

**Conclusion:**

Prevalence of personal and work-related burnout in Australian midwives was high. The physical and psychological exhaustion associated with the different types of burnout were reflected in symptoms of depression, anxiety and stress symptoms. Further research is needed to support the personal well-being of midwives and minimize workplace burnout by developing short and long term strategies.

**Electronic supplementary material:**

The online version of this article (doi:10.1186/s12884-016-1212-5) contains supplementary material, which is available to authorized users.

## Background

Burnout occurs as a result of chronic stress and is characterized by symptoms of emotional and physical fatigue [1]. Some researchers have conceptualised burnout according to potential sources of psychological fatigue such as personal, client, and work related domains [2, 3]. The work of midwives is acknowledged as emotionally demanding [4]. Increasingly, midwives provide care to women experiencing a complex range of anxiety, pain, fear, grief and trauma symptoms [5, 6]. Working closely with distressed women may contribute to a sense of emotional burden in midwives [4]. Furthermore, the wellbeing of midwives may be compromised by a range of workplace and personal stress factors [6]. Given midwives’ role in promoting the perinatal health of mothers [7], the well-being of the midwifery workforce warrants close and regular scrutiny.

The prevalence of moderate to high burnout in midwives varies from 20 to 59% in countries such as Sweden [8]; Norway [9], United Kingdom (UK) [10–12]; and Australia [6, 13, 14]. Some researchers have found associations between burnout in midwives and conditions such as traumatic stress [12, 15]; self-appraisal [11]; positive and negative states [15], emotional intelligence [16], as well as stress symptoms [17]. Less attention has been given to relationships between burnout, depression and anxiety. Depression and anxiety are well-established co-morbid conditions with anxiety often contributing to the development of depression [18]. Furthermore, the interplay of burnout and other psychological conditions in the midwifery workforce is under-researched. It is unclear, if burnout has unique aetiology, if burnout precipitates depression and anxiety, or if burnout is a consequence of common depressive symptoms such as fatigue, self-deprecation, negative thoughts and social withdrawal [19, 20].

Research on burnout and psychological morbidity has predominantly been conducted with nurses and other health professionals. Iakovides et al. [21] found a weak relationship between burnout and depression in Greek nurses. They argued that depression was a pervasive disorder affecting most aspects of life whereas burnout tended to be restricted to nurses’ professional environment [21]. Although distinct conditions, symptoms of more severe burnout mimic major depression [21].

In a large and more recent study of teachers (*n* = 1386) across 18 states of North America, Schonfeld and Bainchi [22] argued that burnout was a type of depression, given the large overlap between burnout, depression and anxiety symptoms. Conversely, a large longitudinal cohort study of Finnish dentists (*n* = 2555) reported that burnout predicted psychological morbidity and highlighted the need for differential diagnosis between burnout and depression in the health workforce [23, 24].

In one of the few international comparative studies, Tourigny et al. [19] surveyed nurses from Japan (*n* = 239) and mainland China (*n* = 550) using the Maslach Burnout Inventory (MBI) and the Center for Epidemiological Studies Depression Survey – 10 item (CESD-10) with responses on a 4-point scale of 0–3. The mean CESD item scores reflected similar high rates of depressive symptoms across both countries (2.08 for Japanese nurses and 2.06 for Chinese nurses) and consistent correlations with MBI domains.

### The measurement of burnout

Given the current study aims to explore the inter-relationship of burnout, depression, anxiety and stress symptoms in practising midwives, careful consideration needed to be given to the selection of a burnout measure. The theoretical conceptualization of burnout influences measurement, interpretation, and how the interplay of burnout symptoms with other psychological conditions are understood. The MBI is the dominant scale in the field but is only commercially available [1, 25]. MBI aims to measure the experiences of individuals who work in the human services sector and reflects symptoms related to emotional exhaustion, depersonalization, and reduced personal accomplishment [25]. However, other researchers argue that the domains of depersonalization and personal accomplishment do not pertain to burnout [2, 3]. A circular, causal relationship between the emotional burden of working with clients and burnout is also implied in the MBI [2, 26]. Furthermore, it is unclear if burnout, as reflected in MBI items, is a ‘state’ of being, a coping strategy, or an effect [3].

In contrast, the CBI subscales of personal, client, and work related burnout reflect an overarching concept of emotional and physical exhaustion according to its source and causality, without introducing concepts of depersonalisation and personal accomplishment [2, 3]. When comparing the MBI and CBI scales, Winwood and Winefield [26] found the CBI (1) accurately conceptualized burnout as a fatigue phenomenon, (2) had good reliability and validity, (3) distinguished between work and personal factors, and (4) was suitable for use with health professionals because of the inclusion of client-related burnout. The CBI was therefore chosen in the current study because of its focus on the “source” of stress as opposed to symptoms. The CBI was also seen as relevant to midwifery workforce research by explicitly asking about work and client related burnout. Furthermore, the CBI has the potential to inform our understanding of the interplay of burnout and depression symptoms in midwives by distinguishing work and non-work sources of stress.

### Rationale for the study

The adverse consequences of burnout and psychological morbidity can be significant. In a literature review on mental illness, burnout and medical errors, Brown et al. [27] determined that many doctors suffering burnout have co-morbidities such as depression and substance dependency. Sentinel adverse events in practice were deemed to be secondary to the primary cause of undiagnosed, untreated depression and stress disorders, highlighting the need to clearly identify the underlying aetiology of health professionals’ emotional health difficulties in order to give appropriate assistance.

Internationally, there is considerable interest in the relationship between burnout and depression with other workforce populations such as nurses [19, 21], doctors [24], and teachers [22], but studies involving midwives are lacking. Although much of the burnout literature refers to workplace stress as a contributor to burnout, the contribution of symptoms of psychological ill-health are rarely taken into consideration.

Significant legislative changes to maternity services in Australia [28] and ongoing calls for global health system change to enable midwives to work to their full scope of practice [29] underpin the urgent need to investigate the psychological well-being of midwives as important primary health care providers. This study aimed to investigate the prevalence of burnout in a population cohort of Australian midwives and interactions with symptoms of depression, anxiety and stress. This Australian data was collected as part of an international collaboration of midwifery researchers in the Work, Health and Emotional Life of Midwives (WHELM) study (Additional file 1).

## Method

### Design

A cross sectional survey design was used.

### Sample and recruitment

Approximately 4600 registered midwives in Australia were invited to participate via an email from the Australian College of Midwives and through informal networks. The email included an invitation outlining the aims of the study, contact details of researchers and a live link to the host survey platform (Qualtrics) [30]. Completed surveys were given identifiers for tracking purposes only.

### Measures

The full survey included demographic questions and several validated scales related to the workplace and midwives’ sense of empowerment. This paper reports on findings from two scales, the Copenhagen Burnout Inventory (CBI), and Depression, Anxiety and Stress Scale (DASS). The CBI comprises three subscales: personal (six items), work burnout (seven items), and client burnout (six items). Twelve items have responses of frequency along a five point Likert scale ranging from ‘100 (always), 75 (often), 50 (sometimes), 25 (seldom) and 0 (never/almost never). Seven items use response categories according to intensity ranging from ‘a very low degree’ to ‘to a very high degree’ [3]. Typical items are: “*how often do you feel tired*”, “*do you feel burnt out because of your work*” and “*do you find it hard to work with clients*”. Scores of 50 to 74 are considered ‘moderate’, 75–99 are high, and a score of 100 is considered severe burnout. All items are straightforward, positively skewed, relate to the relevant subscale and have high internal reliability [3]. In the current study, Cronbach alpha reliability coefficients of the CBI subscales were high (personal α = .90; work-related α = .88; and client-related α = .89).

The Depression, Anxiety and Stress Scale-21 items [31] has three subscales assessing anxiety (7 items), depression (7 items) and stress (7 items). Example items include “*I felt I was using a lot of nervous energy*” (anxiety subscale), “*I tended to over-react to situations*” (depression subscale) and “*I found it hard to wind down*” (stress subscale). The DASS-21 shows good reliability, as well as discriminant and convergent validity with other scales designed to assess depression and anxiety [32]. In the current study, Cronbach alpha reliability coefficients for each subscale were also good (depression α = .91; anxiety α = .85; and stress α = .88).

### Statistical analyses

Descriptive statistics for the sample, CBI, and DASS subscales were generated. As DASS scores were skewed, non-parametric Spearman’s rho was used to identify correlations between CBI and DASS subscales. DASS subscale scores were used as continuous variables, and also collapsed into two groups (normal/mild versus moderate/severe/extremely severe) using cut points provided in the DASS User’s Manual [31]. Mann-Whitney U tests compared burnout scores across the two clinical groups. Effect size statistics were also calculated (*r* = z/square root of N), and Cohen’s [33] guidelines used to judge the size of the effect (small = .1, medium = .3, large = .5). If a case had any missing scale item responses, it was excluded from the analysis.

### Ethical considerations

Human research ethics approval was obtained from Griffith University (NRS/39/11/HREC). Consent was implied through completion of the survey. Anonymity of participants was assured as no name related data was collected.

## Results

### Characteristics of midwives

A total of 1037 survey forms were received giving an estimated response rate of around 22.5%. Respondents were predominantly female (98%), with an average age of 46.43 years, and 16.51 years of practice.

### Rates of burnout, depression, anxiety and stress

Mean CBI subscale scores for this sample were 55.9 (personal); 48.4 (work-related) and 25.6 (client-related). However, it is more meaningful to inspect the overall prevalence of midwives reporting moderate or higher burnout. On the personal subscale 46.1% (*n* = 456 out of 990 cases) of midwives reported moderate burnout, 17.4% (*n* = 173) reported high burnout and 14 (1.4%) participants scored 100 indicating severe personal burnout. The overall prevalence of midwives reporting moderate work-related burnout was 36.4% (*n* = 356 out of 978 cases). Seventy-one (7.3%) midwives reported high levels and one respondent had severe work-related burnout. The overall prevalence of midwives reporting moderate or higher client-related burnout was very low at 10.4% (*n* = 102 out of 984 cases). Ten (1%) midwives reported high levels and one had severe client-related burnout.

The majority of midwives (between 78 and 83%) scored in the normal-mild range on the DASS subscales. But around 20% of midwives reported moderate/severe/extreme levels of depression (17.3%); anxiety (20.4%), and stress (22.1%) symptoms. The proportion of midwives recording DASS scores in the moderate/severe/ extremely severe ranges are shown in Table 1.

**Table 1:** Scores, cut-offs and reliability of the Copenhagen Burnout Inventory and Depression, Anxiety and Stress Scales

Measure	M (SD)	Prevalence cut-off	Subscale Cronbach alpha
		N (%)	
CBI			
Personal Burnout *N* = 990	55.90 (18.06)	No/low (<50) = 347 (35.1)	.91
		Moderate (50–74) = 456 (46.1)	
		High (75–99) = 173 (17.4)	
		Severe (100) = 14 (1.4)	
Work Burnout *N* = 978	44.69 (19.23)	No/low (<50) = 550 (56.2)	.88
		Moderate (50–74) = 356 (36.4)	
		High (75–99) = 71 (7)	
		Severe (100) = 1 (.1)	
Client-related Burnout *N* = 984	19.32 (19.22)	No/low (<50) = 882 (89.6)	.89
		Moderate (50–74) = 90 (9.2)	
		High (75–99) = 10 (1.0)	
		Severe (100) = 2 (0.2)	
DASS			
Depression	6.66 (8.46)	Normal/mild = 807 (83)	.91
		Mod/severe/extreme = 169 (17)	
Anxiety	5.35 (6.92)	Normal/mild = 777 (79.5)	.85
		Mod/severe/extreme = 200 (20.5)	
Stress	11.13 (8.91)	Normal/mild = 762 (78)	.88
		Mod/severe/extreme = 241 (22)	

*CBI* Copenhagen Burnout Inventory, *DASS-21* Depression, Anxiety and Stress Scale, *M* Mean, *SD* standard deviation

All burnout subscales were significantly correlated with depression, anxiety and stress, particularly personal and work-related burnout with Spearman’s rho correlations ranging from .51 to .63 (*p* < .001) as shown in Table 2. A series of Mann-Whitney U Tests revealed significant differences in burnout levels of midwives with normal/mild depression (Md = 39.28, *n* = 800) compared to those with moderate/severe/extreme depression (Md = 60.71, *n* = 168) (U = 23,281, z = −13.35, *p* < .001, *r* = .43); normal/mild anxiety (Md = 39.28, *n* = 771) compared to those with moderate/severe/extreme anxiety (Md = 60.71, *n* = 198) (U = 31,661, z = −12.75, *p* < .001, *r* = .41); and normal/mild stress (Md = 39.28, *n* = 754) compared to those with moderate/severe/extreme stress (Md = 62.5, *n* = 214) (U = 26388.5, z = −15.06, *p* < .001, *r* = .48).

**Table 2:** Spearman’s rho correlations between Copenhagen Burnout Inventory and Depression, Anxiety and Stress Scales

		DASS Dep	DASS Anx	DASS Stress
CBI personal	Correlation coefficient	.62^a^	.51^a^	.59^a^
	N	975	975	975
CBI work-related	Correlation coefficient	.63^a^	.53^a^	.63^a^
	N	968	969	968
CBI client-related	Correlation coefficient	.39^a^	.31	.39^a^
	N	974	975	974

^a^Correlation is significant at the 0.01 level (2-tailed)

## Discussion

This large cross-sectional survey of Australian midwives revealed a high level of personal and work-related burnout. Furthermore, around 20% of midwives reported significant symptoms of depression, anxiety and stress. No previous study has reported on the correlation of burnout with symptoms of depression, anxiety and stress in the midwifery workforce. In order to compare our results with other Australian and international studies, subscale means were reported (Personal mean 55.8; Work mean 44.6; Client-related mean 19.3). The personal subscale burnout mean was comparable with earlier surveys of midwives (*n* = 58) working in a maternity unit in Queensland, Australia which reported a personal burnout mean of 52.1 [13]. Conversely, our results are much higher than those reported in a comparison of work satisfaction and burnout levels between caseload (*n* = 20) and standard care midwives (*n* = 130) in Victoria, Australia [14]. Although these caseload midwives reported considerably lower personal burnout mean scores; work-related burnout, and client-related burnout at baseline; burnout mean scores of midwives working in standard care models were comparable to results reported in our study.

These conflicting results may reflect changing workplace circumstances for each study. Jordan et al. [13] reported that their respondents were experiencing a great deal of stressful organizational change, whereas caseload midwives in Victoria may have been excited by the implementation of the new caseload roles at that time. Indeed, at the 2-year follow-up, caseload midwives reported even lower CBI subscale mean scores which continued to be significantly lower than midwives working in the standard care model [14].

Our CBI subscale mean scores were also considerably higher than those reported by Scandinavian researchers. A recent survey in Norway by Henriksen and Lukasse [9] found that only 20% of midwives reported symptoms of personal and work-related burnout. This is much lower than the 42.9% rate of personal burnout reported by Swedish researchers [8]. Rates of personal burnout for participating Norwegian and Swedish midwives were also low compared to the small cohort of Danish midwives (*n* = 41) who participated in a prospective longitudinal study of human sector employees known as the Project on Burnout, Motivation and Job Satisfaction (PUMA) [3, 34]. Compared to our findings, the Danish sample reported less personal burnout (mean 44.7), similar levels of work-related burnout (mean 43.5), but higher client-related burnout (38.4).

Hildingsson et al. [8] suggested that the rates of personal burnout in Swedish midwives may reflect their lack of autonomy in the obstetric-driven approach to care. Other researchers have concluded that caseload midwives experience high levels of satisfaction from working in continuity of care models which foster close relationships with women, occupational autonomy, rewards personal investment, and enables midwives to make a positive difference to women [14, 35].

The proportion of Australian midwives reporting moderate or higher burnout was significantly greater than any other published study to date. Even though Hildingsson et al. [8] refers to “high” burnout, a breakdown of proportions was not reported, and the relatively low mean scores suggest that the prevalence of moderate personal burnout (39.5%) was being reported. Even so, fewer Swedish midwives reported burnout than the 64.9% of Australian midwives reporting moderate or higher burnout here.

High rates of burnout reported by Australian midwives may relate to work and client factors in the dominant fragmented, medical-led maternity care system [28]. While there is an increasing trend towards midwifery continuity of care (caseload) across pregnancy, labour and birth and postpartum, access to these models of care are still limited [28, 36]. Workplace stress experienced by hospital-based midwives seems to be consistent in comparative studies (e.g., 14). Banovcinova and Baskova [35] reported that hospital-based midwives in shift work models were more likely to report harrassment, abuse, or bullying from medical and midwifery colleagues which potentially contributed to higher levels of burnout (on the MBI) compared to midwives working in the community. The stress associated with interpersonal professional conflict has been viewed as both a primary contributor to burnout and as well as a consequence of burnout [6] and deserves further investigation. Research on burnout also needs to be understood in relation to different work contexts of participating midwives [37]. Unlike previous studies, Henriksen and Lukasse [9] found shift work, hours worked and workload contributed little to burnout. Future research could consider mixed method approaches to understand the worklife experiences of midwives through interviews as well as large cohort surveys.

### Depression, anxiety and stress

Around 20% of midwives in our sample reported severe levels of depression, anxiety and stress symptoms. The only other Australian study with midwives and nurses using the DASS sought to test the effectiveness of a mindfulness-based stress reduction program on mental health outcomes [38]. At baseline, these midwives (*n* = 20) and nurses (*n* = 20) who volunteered for the program reported similar mean scores on the depression, anxiety and stress subscales as those reported by us. Although the pilot intervention was successful in improving the emotional wellbeing of participants, levels of depression were not significantly affected after the 8-week intervention [38].

Our results suggest the impact of burnout, depression, anxiety, and stress symptoms is pervasive across midwives’ personal and work lives. There was a consistent correlation between the sense of physical and emotional exhaustion reflected in the personal burnout subscale and symptoms of depression and anxiety. While there is increasing understanding about aspects of work that contribute to burnout [6, 8–10, 35], less is known if feelings of dissatisfaction, lack of control, and learned helplessness pervade personal and work spheres and contribute simultaneously to burnout and depression. Future research could use a prospective, longitudinal design in order to follow a cohort of midwives to determine if occupational burnout proceeds depression or if personal factors affecting women (such as stress of child-rearing, or being in a violent intimate relationship) contributes to depression and anxiety and impact on well-being at work.

Midwives have reported high levels of stress due to the nature of their work, workplace conditions, and their socialization into ways of working that minimize self-care [38, 39]. Researchers suggest that workplace circumstances contribute to some midwives becoming highly susceptible to occupational burnout, which eventually contributes to reduced quality of care. For example, the high rates of emotional exhaustion (according to the MBI) reported by UK midwives was suggested to reflect stress associated with changes to the maternity services in that country [11]. Furthermore, as our findings suggest, workplace burnout can have significant implications for the development of other mental health conditions such as depression and anxiety. Furthermore, Sheen, Spiby and Slade [12] identified that UK midwives who reported trauma symptoms were also likely to have high levels of burnout symptoms. The nature and organisation of midwifery work therefore needs to be considered in the development of prevention or management interventions.

#### Limitations

Our findings need to be considered in light of possible limitations. Although a relatively large number of midwives responded, it could be that this survey may have been completed by midwives most interested in this topic. Some midwives experiencing severe burnout or depression may not have participated and the findings may be an under-estimation of the true extent of these conditions in the midwifery workforce. Conversely, it could be that midwives experiencing burnout were more interested in completing the survey and the results may be an over-estimate. The cross-sectional design does not permit cause and effect to be concluded, but does highlight prevalence and relationships amongst factors as the basis for future research.

#### Implications for research and practice

CBI and DASS were found to be reliable, valid tools to measure mental health risk in the midwifery workforce. Measures of burnout and mental health symptoms need to be used sensitively in research and in practice when assessing the well-being of the workforce. Confidentiality of any mental health self-assessment results need to be maintained to reduce stigma and discrimination towards individuals in the workplace. Self-assessment or disclosure of mental health concerns need to be managed positively. Burnout, depression, anxiety and stress symptoms can be acknowledged in the workplace, contributing factors addressed, impact minimised, and effective treatments implemented.

Emotional well-being of the midwifery workforce has considerable implications for safe maternity care, staff retention and maternal satisfaction. If midwives suffer emotional exhaustion, they are likely to be emotionally withdrawn and communicate poorly with women, their families and colleagues; distance themselves from women in their care; and perform poorly in the workplace [40]. Interventions to minimize burnout and promote well-being and resilience are gaining greater attention. The mindfulness intervention by Foureur et al. [38] found improvements in participants’ general health, sense of coherence and lowered stress but not depression. It could be that exercise and lifestyle interventions may be more efficacious than psychological intervention programs for addressing depression and burnout.

Midwives experiencing excessive stress and early-to-moderate burnout may benefit from a combination of approaches, including exercise, one-on-one coaching, reflective clinical supervision, coping skills/strategies, addressing what can be modified in the workplace environment, and strategies emphasizing work-life balance [41]. Midwives experiencing severe burnout may require, in addition, a temporary leave of absence, psychiatric evaluation and treatment for major depression and other comorbid diagnoses, as well as a supportive return-to-work program that fosters autonomy, team work, and work satisfaction. There is also evidence by other researchers that enabling midwives to work in caseload models contributes to better emotional wellbeing [14] and feelings of professional safety [10, 39] suggesting this model should be more widely adopted.

## Conclusions

Prevalence of moderate to severe personal and work-related burnout, depression, anxiety and stress in Australian midwives was high. Responses on the CBI were strong predictors of mental health distress in midwives. Exploring levels of burnout, depression, anxiety and stress within the midwifery population is important to better understand and address the development of responses to the stressful nature of their work and workplace demands.

## Additional file

Additional file 1: WHELM Dataset_Aust_16Feb2016. (DOCX 158 kb)

## Abbreviations

CBI: Copenhagen Burnout Inventory
CESD-10: Center for Epidemiological Studies Depression Survey – 10 item
DASS: Depression, Anxiety and Stress Scale
MBI: Maslach Burnout Inventory
PUMA: Project on Burnout, Motivation and Job Satisfaction
UK: United Kingdom
WHO: World Health Organization
