# Supplementary material for: Prevalence of burnout, depression, anxiety and stress in Australian midwives: a cross-sectional survey
Source: BMC Pregnancy Childbirth. 2017 Jan 9;17:13. doi: 10.1186/s12884-016-1212-5 (PMC5223536; doi:10.1186/s12884-016-1212-5)
Supplement: Additional file 1: — WHELM Dataset_Aust_16Feb2016. (DOCX 158 kb) [file 12884_2016_1212_MOESM1_ESM.docx]

**WHELM – Australian dataset 16 Feb 2016**

Contents

[Characteristics of the sample 2](#_Toc445879137)

[Gender 2](#_Toc445879138)

[Registration status 2](#_Toc445879139)

[Principal role 2](#_Toc445879140)

[DASS subscales descriptives 3](#_Toc445879141)

[Scores on DASS subscales 3](#_Toc445879142)

[DASS Summary statistics 3](#_Toc445879143)

[Stress 3](#_Toc445879144)

[Anxiety 4](#_Toc445879145)

[Depression 4](#_Toc445879146)

[DASS Histograms 5](#_Toc445879147)

[DASS Clinical groups 7](#_Toc445879148)

[DASS subscales collapsed into 2 groups (normal, mild/ mod, severe, extreme) 8](#_Toc445879149)

[Burnout descriptives 9](#_Toc445879150)

[Burnout Histograms 11](#_Toc445879151)

[Reliability 13](#_Toc445879152)

[Reliability DASS 13](#_Toc445879153)

[DASS Stress 13](#_Toc445879154)

[DASS Anxiety 14](#_Toc445879155)

[DASS Depression 15](#_Toc445879156)

[Reliability Burnout 16](#_Toc445879157)

[Personal burnout 16](#_Toc445879158)

[Burnout work 17](#_Toc445879159)

[Burnout Client 19](#_Toc445879160)

[Association between burnout and DASS subscales 21](#_Toc445879161)

[Spearman correlations between Burnout and DASS 21](#_Toc445879162)

[Boxplots of Burnout-Work with DASS in 2 groups 21](#_Toc445879163)

[Anxiety 21](#_Toc445879164)

[Depression 22](#_Toc445879165)

[Stress 22](#_Toc445879166)

[Compare Burnout scores across DASS clinical 2 groups 23](#_Toc445879167)

[Stress 23](#_Toc445879168)

[Depression 23](#_Toc445879169)

[Anxiety 24](#_Toc445879170)

# Characteristics of the sample

### Gender

| **Q4 Gender** | | | | | |
| --- | --- | --- | --- | --- | --- |
|  | | Frequency | Percent | Valid Percent | Cumulative Percent |
| Valid | 1 Female | 1017 | 98.1 | 98.1 | 98.1 |
|  | 2 Male | 20 | 1.9 | 1.9 | 100.0 |
|  | Total | 1037 | 100.0 | 100.0 |  |

### Registration status

| **Q16 What is your current registration status?** | | | | | |
| --- | --- | --- | --- | --- | --- |
|  | | Frequency | Percent | Valid Percent | Cumulative Percent |
| Valid | 1 Midwife only | 208 | 20.1 | 20.1 | 20.1 |
|  | 2 Nurse only | 2 | .2 | .2 | 20.3 |
|  | 3 Midwife and nurse | 827 | 79.7 | 79.7 | 100.0 |
|  | Total | 1037 | 100.0 | 100.0 |  |

### Principal role

| **Q32 What is the principal role in your main job in midwifery?** | | | | | |
| --- | --- | --- | --- | --- | --- |
|  | | Frequency | Percent | Valid Percent | Cumulative Percent |
| Valid | 1 Clinician | 846 | 81.6 | 81.6 | 81.6 |
|  | 2 Clinical manager | 65 | 6.3 | 6.3 | 87.8 |
|  | 3 Administrator/ senior manager | 28 | 2.7 | 2.7 | 90.5 |
|  | 4 Teacher / lecturer or clinical educator/facilitator | 86 | 8.3 | 8.3 | 98.8 |
|  | 5 Researcher | 12 | 1.2 | 1.2 | 100.0 |
|  | Total | 1037 | 100.0 | 100.0 |  |

# DASS subscales descriptives

## Scores on DASS subscales

### DASS Summary statistics

| **Statistics** | | | | |
| --- | --- | --- | --- | --- |
|  | | DASSStress | DASSAnx | DASSDep |
| N | Valid | 976 | 977 | 976 |
|  | Missing | 61 | 60 | 61 |
| Mean | | 11.13 | 5.35 | 6.66 |
| Median | | 10.00 | 2.00 | 4.00 |
| Std. Deviation | | 8.911 | 6.922 | 8.459 |
| Minimum | | 0 | 0 | 0 |
| Maximum | | 42 | 42 | 42 |

### Stress

| **DASSStress** | | | | | |
| --- | --- | --- | --- | --- | --- |
|  | | Frequency | Percent | Valid Percent | Cumulative Percent |
| Valid | 0 | 93 | 9.0 | 9.5 | 9.5 |
|  | 2 | 93 | 9.0 | 9.5 | 19.1 |
|  | 4 | 99 | 9.5 | 10.1 | 29.2 |
|  | 6 | 79 | 7.6 | 8.1 | 37.3 |
|  | 8 | 113 | 10.9 | 11.6 | 48.9 |
|  | 10 | 80 | 7.7 | 8.2 | 57.1 |
|  | 12 | 82 | 7.9 | 8.4 | 65.5 |
|  | 14 | 77 | 7.4 | 7.9 | 73.4 |
|  | 16 | 46 | 4.4 | 4.7 | 78.1 |
|  | 18 | 45 | 4.3 | 4.6 | 82.7 |
|  | 20 | 29 | 2.8 | 3.0 | 85.7 |
|  | 22 | 21 | 2.0 | 2.2 | 87.8 |
|  | 24 | 30 | 2.9 | 3.1 | 90.9 |
|  | 26 | 22 | 2.1 | 2.3 | 93.1 |
|  | 28 | 23 | 2.2 | 2.4 | 95.5 |
|  | 30 | 11 | 1.1 | 1.1 | 96.6 |
|  | 32 | 7 | .7 | .7 | 97.3 |
|  | 34 | 7 | .7 | .7 | 98.1 |
|  | 36 | 8 | .8 | .8 | 98.9 |
|  | 38 | 4 | .4 | .4 | 99.3 |
|  | 40 | 3 | .3 | .3 | 99.6 |
|  | 42 | 4 | .4 | .4 | 100.0 |
|  | Total | 976 | 94.1 | 100.0 |  |
| Missing | System | 61 | 5.9 |  |  |
| Total | | 1037 | 100.0 |  |  |

### Anxiety

| **DASSAnx** | | | | | |
| --- | --- | --- | --- | --- | --- |
|  | | Frequency | Percent | Valid Percent | Cumulative Percent |
| Valid | 0 | 313 | 30.2 | 32.0 | 32.0 |
|  | 2 | 187 | 18.0 | 19.1 | 51.2 |
|  | 4 | 129 | 12.4 | 13.2 | 64.4 |
|  | 6 | 83 | 8.0 | 8.5 | 72.9 |
|  | 8 | 65 | 6.3 | 6.7 | 79.5 |
|  | 10 | 46 | 4.4 | 4.7 | 84.2 |
|  | 12 | 33 | 3.2 | 3.4 | 87.6 |
|  | 14 | 28 | 2.7 | 2.9 | 90.5 |
|  | 16 | 22 | 2.1 | 2.3 | 92.7 |
|  | 18 | 12 | 1.2 | 1.2 | 94.0 |
|  | 20 | 17 | 1.6 | 1.7 | 95.7 |
|  | 22 | 7 | .7 | .7 | 96.4 |
|  | 24 | 13 | 1.3 | 1.3 | 97.7 |
|  | 26 | 5 | .5 | .5 | 98.3 |
|  | 28 | 2 | .2 | .2 | 98.5 |
|  | 30 | 6 | .6 | .6 | 99.1 |
|  | 32 | 2 | .2 | .2 | 99.3 |
|  | 34 | 2 | .2 | .2 | 99.5 |
|  | 36 | 1 | .1 | .1 | 99.6 |
|  | 38 | 1 | .1 | .1 | 99.7 |
|  | 42 | 3 | .3 | .3 | 100.0 |
|  | Total | 977 | 94.2 | 100.0 |  |
| Missing | System | 60 | 5.8 |  |  |
| Total | | 1037 | 100.0 |  |  |

### Depression

| **DASSDep** | | | | | |
| --- | --- | --- | --- | --- | --- |
|  | | Frequency | Percent | Valid Percent | Cumulative Percent |
| Valid | 0 | 285 | 27.5 | 29.2 | 29.2 |
|  | 2 | 164 | 15.8 | 16.8 | 46.0 |
|  | 4 | 124 | 12.0 | 12.7 | 58.7 |
|  | 6 | 80 | 7.7 | 8.2 | 66.9 |
|  | 8 | 71 | 6.8 | 7.3 | 74.2 |
|  | 10 | 44 | 4.2 | 4.5 | 78.7 |
|  | 12 | 39 | 3.8 | 4.0 | 82.7 |
|  | 14 | 35 | 3.4 | 3.6 | 86.3 |
|  | 16 | 31 | 3.0 | 3.2 | 89.4 |
|  | 18 | 19 | 1.8 | 1.9 | 91.4 |
|  | 20 | 10 | 1.0 | 1.0 | 92.4 |
|  | 22 | 11 | 1.1 | 1.1 | 93.5 |
|  | 24 | 15 | 1.4 | 1.5 | 95.1 |
|  | 26 | 6 | .6 | .6 | 95.7 |
|  | 28 | 7 | .7 | .7 | 96.4 |
|  | 30 | 8 | .8 | .8 | 97.2 |
|  | 32 | 8 | .8 | .8 | 98.1 |
|  | 34 | 2 | .2 | .2 | 98.3 |
|  | 36 | 4 | .4 | .4 | 98.7 |
|  | 38 | 1 | .1 | .1 | 98.8 |
|  | 40 | 6 | .6 | .6 | 99.4 |
|  | 42 | 6 | .6 | .6 | 100.0 |
|  | Total | 976 | 94.1 | 100.0 |  |
| Missing | System | 61 | 5.9 |  |  |
| Total | | 1037 | 100.0 |  |  |

### DASS Histograms


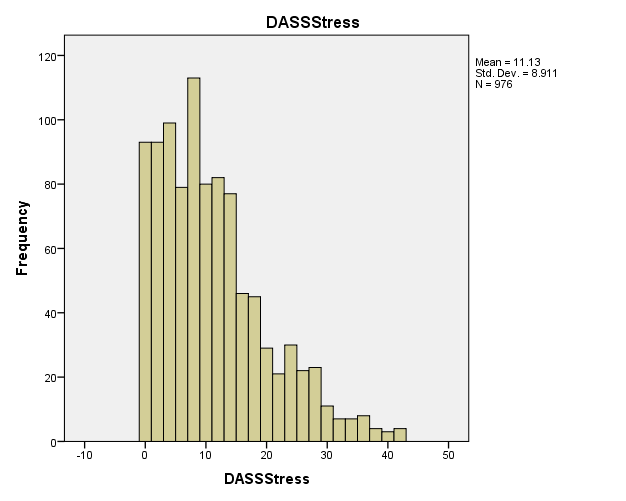


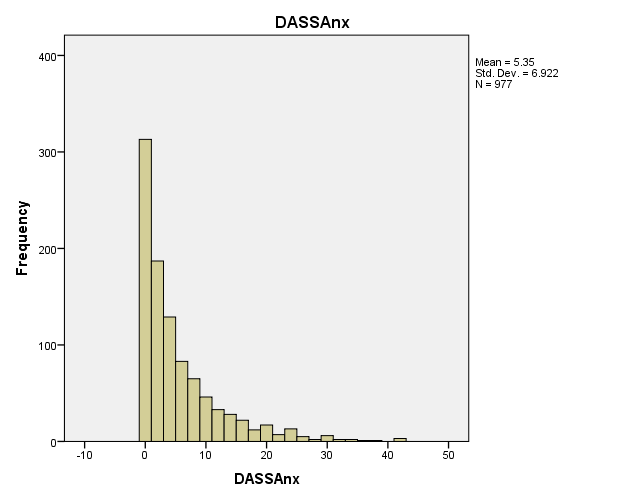


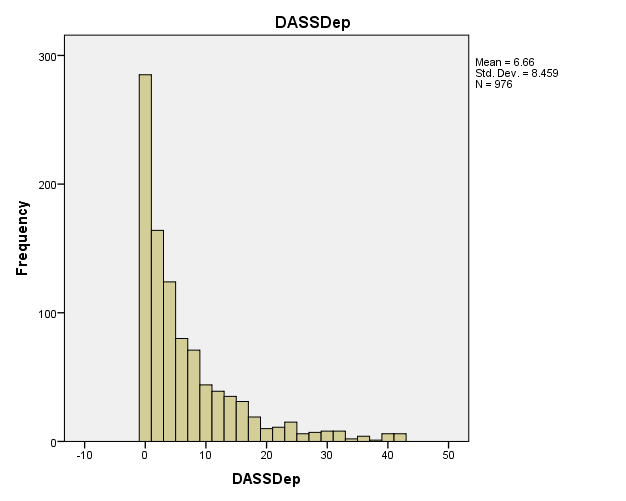


## DASS Clinical groups

| **DASstressGp** | | | | | |
| --- | --- | --- | --- | --- | --- |
|  | | Frequency | Percent | Valid Percent | Cumulative Percent |
| Valid | 1 normal | 716 | 69.0 | 73.4 | 73.4 |
|  | 2 mild | 46 | 4.4 | 4.7 | 78.1 |
|  | 3 moderate | 125 | 12.1 | 12.8 | 90.9 |
|  | 4 severe | 63 | 6.1 | 6.5 | 97.3 |
|  | 5 extremely severe | 26 | 2.5 | 2.7 | 100.0 |
|  | Total | 976 | 94.1 | 100.0 |  |
| Missing | System | 61 | 5.9 |  |  |
| Total | | 1037 | 100.0 |  |  |

| **DASSAnxgp** | | | | | |
| --- | --- | --- | --- | --- | --- |
|  | | Frequency | Percent | Valid Percent | Cumulative Percent |
| Valid | 1 normal | 712 | 68.7 | 72.9 | 72.9 |
|  | 2 mild | 65 | 6.3 | 6.7 | 79.5 |
|  | 3 moderate | 107 | 10.3 | 11.0 | 90.5 |
|  | 4 severe | 34 | 3.3 | 3.5 | 94.0 |
|  | 5 extremely severe | 59 | 5.7 | 6.0 | 100.0 |
|  | Total | 977 | 94.2 | 100.0 |  |
| Missing | System | 60 | 5.8 |  |  |
| Total | | 1037 | 100.0 |  |  |

| **DASSDepgp** | | | | | |
| --- | --- | --- | --- | --- | --- |
|  | | Frequency | Percent | Valid Percent | Cumulative Percent |
| Valid | 1 normal | 724 | 69.8 | 74.2 | 74.2 |
|  | 2 mild | 83 | 8.0 | 8.5 | 82.7 |
|  | 3 moderate | 85 | 8.2 | 8.7 | 91.4 |
|  | 4 severe | 42 | 4.1 | 4.3 | 95.7 |
|  | 5 extremely severe | 42 | 4.1 | 4.3 | 100.0 |
|  | Total | 976 | 94.1 | 100.0 |  |
| Missing | System | 61 | 5.9 |  |  |
| Total | | 1037 | 100.0 |  |  |

## DASS subscales collapsed into 2 groups (normal, mild/ mod, severe, extreme)

| **DASSStressGp2** | | | | | |
| --- | --- | --- | --- | --- | --- |
|  | | Frequency | Percent | Valid Percent | Cumulative Percent |
| Valid | 1 normal/mild | 762 | 73.5 | 78.1 | 78.1 |
|  | 2 mod/severe/extreme | 214 | 20.6 | 21.9 | 100.0 |
|  | Total | 976 | 94.1 | 100.0 |  |
| Missing | System | 61 | 5.9 |  |  |
| Total | | 1037 | 100.0 |  |  |

| **DASSAnxGp2** | | | | | |
| --- | --- | --- | --- | --- | --- |
|  | | Frequency | Percent | Valid Percent | Cumulative Percent |
| Valid | 1 normal/mild | 777 | 74.9 | 79.5 | 79.5 |
|  | 2 mod/severe/extreme | 200 | 19.3 | 20.5 | 100.0 |
|  | Total | 977 | 94.2 | 100.0 |  |
| Missing | System | 60 | 5.8 |  |  |
| Total | | 1037 | 100.0 |  |  |

| **DASSDepGp2** | | | | | |
| --- | --- | --- | --- | --- | --- |
|  | | Frequency | Percent | Valid Percent | Cumulative Percent |
| Valid | 1 normal/mild | 807 | 77.8 | 82.7 | 82.7 |
|  | 2 mod/severe/extreme | 169 | 16.3 | 17.3 | 100.0 |
|  | Total | 976 | 94.1 | 100.0 |  |
| Missing | System | 61 | 5.9 |  |  |
| Total | | 1037 | 100.0 |  |  |

# Burnout descriptives

| **Statistics** | | | | |
| --- | --- | --- | --- | --- |
|  | | TBurn_Personal | TBurn_WorkCorrect | TBurn_ClientCorrect |
| N | Valid | 990 | 978 | 984 |
|  | Missing | 47 | 59 | 53 |
| Mean | | 55.8965 | 44.6940 | 19.3216 |
| Median | | 54.1667 | 42.8571 | 12.5000 |
| Std. Deviation | | 18.05806 | 19.22706 | 19.21783 |
| Minimum | | 8.33 | 3.57 | .00 |
| Maximum | | 100.00 | 100.00 | 100.00 |

| **TBurn_Personal** | | | | | |
| --- | --- | --- | --- | --- | --- |
|  | | Frequency | Percent | Valid Percent | Cumulative Percent |
| Valid | 8.33 | 1 | .1 | .1 | .1 |
|  | 12.50 | 7 | .7 | .7 | .8 |
|  | 16.67 | 11 | 1.1 | 1.1 | 1.9 |
|  | 20.83 | 11 | 1.1 | 1.1 | 3.0 |
|  | 25.00 | 28 | 2.7 | 2.8 | 5.9 |
|  | 29.17 | 29 | 2.8 | 2.9 | 8.8 |
|  | 33.33 | 41 | 4.0 | 4.1 | 12.9 |
|  | 37.50 | 64 | 6.2 | 6.5 | 19.4 |
|  | 41.67 | 70 | 6.8 | 7.1 | 26.5 |
|  | 45.83 | 85 | 8.2 | 8.6 | 35.1 |
|  | 50.00 | 73 | 7.0 | 7.4 | 42.4 |
|  | 54.17 | 78 | 7.5 | 7.9 | 50.3 |
|  | 58.33 | 73 | 7.0 | 7.4 | 57.7 |
|  | 62.50 | 96 | 9.3 | 9.7 | 67.4 |
|  | 66.67 | 70 | 6.8 | 7.1 | 74.4 |
|  | 70.83 | 66 | 6.4 | 6.7 | 81.1 |
|  | 75.00 | 83 | 8.0 | 8.4 | 89.5 |
|  | 79.17 | 35 | 3.4 | 3.5 | 93.0 |
|  | 83.33 | 17 | 1.6 | 1.7 | 94.7 |
|  | 87.50 | 22 | 2.1 | 2.2 | 97.0 |
|  | 91.67 | 10 | 1.0 | 1.0 | 98.0 |
|  | 95.83 | 6 | .6 | .6 | 98.6 |
|  | 100.00 | 14 | 1.4 | 1.4 | 100.0 |
|  | Total | 990 | 95.5 | 100.0 |  |
| Missing | System | 47 | 4.5 |  |  |
| Total | | 1037 | 100.0 |  |  |

| **TBurn_WorkCorrect** | | | | | |
| --- | --- | --- | --- | --- | --- |
|  | | Frequency | Percent | Valid Percent | Cumulative Percent |
| Valid | 3.57 | 7 | .7 | .7 | .7 |
|  | 7.14 | 14 | 1.4 | 1.4 | 2.1 |
|  | 10.71 | 24 | 2.3 | 2.5 | 4.6 |
|  | 14.29 | 22 | 2.1 | 2.2 | 6.9 |
|  | 17.86 | 41 | 4.0 | 4.2 | 11.0 |
|  | 21.43 | 29 | 2.8 | 3.0 | 14.0 |
|  | 25.00 | 53 | 5.1 | 5.4 | 19.4 |
|  | 28.57 | 51 | 4.9 | 5.2 | 24.6 |
|  | 32.14 | 60 | 5.8 | 6.1 | 30.8 |
|  | 35.71 | 60 | 5.8 | 6.1 | 36.9 |
|  | 39.29 | 78 | 7.5 | 8.0 | 44.9 |
|  | 42.86 | 57 | 5.5 | 5.8 | 50.7 |
|  | 46.43 | 54 | 5.2 | 5.5 | 56.2 |
|  | 50.00 | 66 | 6.4 | 6.7 | 63.0 |
|  | 53.57 | 64 | 6.2 | 6.5 | 69.5 |
|  | 57.14 | 62 | 6.0 | 6.3 | 75.9 |
|  | 60.71 | 53 | 5.1 | 5.4 | 81.3 |
|  | 64.29 | 46 | 4.4 | 4.7 | 86.0 |
|  | 67.86 | 41 | 4.0 | 4.2 | 90.2 |
|  | 71.43 | 24 | 2.3 | 2.5 | 92.6 |
|  | 75.00 | 17 | 1.6 | 1.7 | 94.4 |
|  | 78.57 | 22 | 2.1 | 2.2 | 96.6 |
|  | 82.14 | 14 | 1.4 | 1.4 | 98.1 |
|  | 85.71 | 10 | 1.0 | 1.0 | 99.1 |
|  | 89.29 | 3 | .3 | .3 | 99.4 |
|  | 92.86 | 3 | .3 | .3 | 99.7 |
|  | 96.43 | 2 | .2 | .2 | 99.9 |
|  | 100.00 | 1 | .1 | .1 | 100.0 |
|  | Total | 978 | 94.3 | 100.0 |  |
| Missing | System | 59 | 5.7 |  |  |
| Total | | 1037 | 100.0 |  |  |

| **TBurn_ClientCorrect** | | | | | |
| --- | --- | --- | --- | --- | --- |
|  | | Frequency | Percent | Valid Percent | Cumulative Percent |
| Valid | .00 | 245 | 23.6 | 24.9 | 24.9 |
|  | 4.17 | 96 | 9.3 | 9.8 | 34.7 |
|  | 8.33 | 97 | 9.4 | 9.9 | 44.5 |
|  | 12.50 | 61 | 5.9 | 6.2 | 50.7 |
|  | 16.67 | 52 | 5.0 | 5.3 | 56.0 |
|  | 20.83 | 50 | 4.8 | 5.1 | 61.1 |
|  | 25.00 | 84 | 8.1 | 8.5 | 69.6 |
|  | 29.17 | 61 | 5.9 | 6.2 | 75.8 |
|  | 33.33 | 47 | 4.5 | 4.8 | 80.6 |
|  | 37.50 | 33 | 3.2 | 3.4 | 83.9 |
|  | 41.67 | 29 | 2.8 | 2.9 | 86.9 |
|  | 45.83 | 27 | 2.6 | 2.7 | 89.6 |
|  | 50.00 | 35 | 3.4 | 3.6 | 93.2 |
|  | 54.17 | 23 | 2.2 | 2.3 | 95.5 |
|  | 58.33 | 13 | 1.3 | 1.3 | 96.8 |
|  | 62.50 | 8 | .8 | .8 | 97.7 |
|  | 66.67 | 10 | 1.0 | 1.0 | 98.7 |
|  | 70.83 | 1 | .1 | .1 | 98.8 |
|  | 75.00 | 3 | .3 | .3 | 99.1 |
|  | 79.17 | 2 | .2 | .2 | 99.3 |
|  | 83.33 | 3 | .3 | .3 | 99.6 |
|  | 87.50 | 1 | .1 | .1 | 99.7 |
|  | 91.67 | 1 | .1 | .1 | 99.8 |
|  | 100.00 | 2 | .2 | .2 | 100.0 |
|  | Total | 984 | 94.9 | 100.0 |  |
| Missing | System | 53 | 5.1 |  |  |
| Total | | 1037 | 100.0 |  |  |

## Burnout Histograms


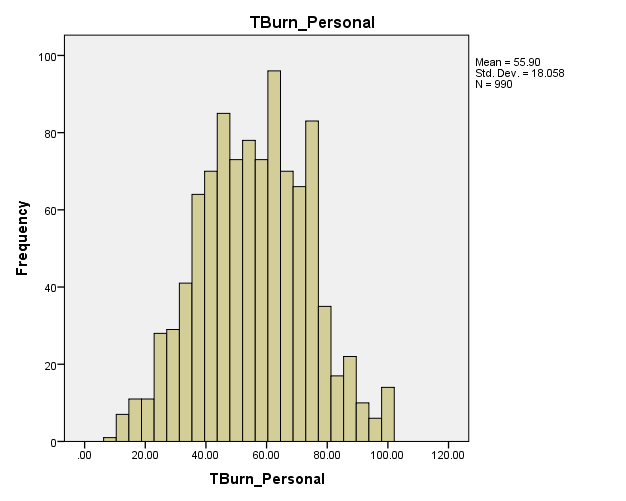


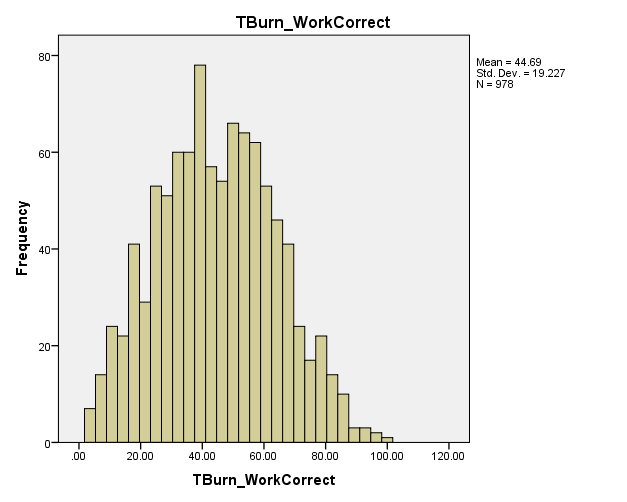


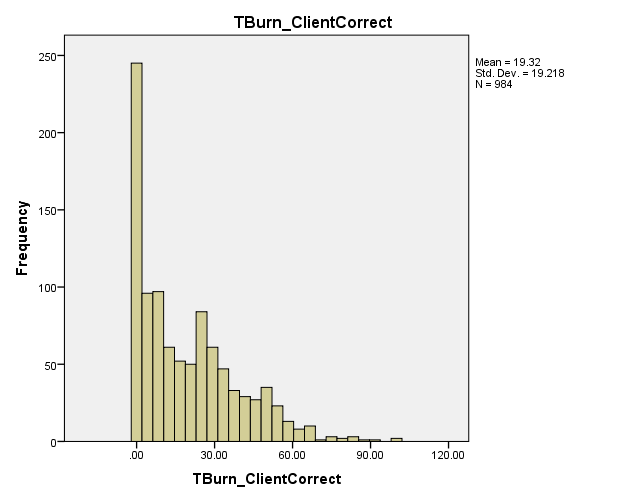


# Reliability

## Reliability DASS

### DASS Stress

| **Case Processing Summary** | | | |
| --- | --- | --- | --- |
|  | | N | % |
| Cases | Valid | 976 | 94.1 |
|  | Excluded^a^ | 61 | 5.9 |
|  | Total | 1037 | 100.0 |
| a. Listwise deletion based on all variables in the procedure. | | | |

| **Reliability Statistics** | | |
| --- | --- | --- |
| Cronbach's Alpha | Cronbach's Alpha Based on Standardized Items | N of Items |
| .883 | .886 | 7 |

| **Item Statistics** | | | |
| --- | --- | --- | --- |
|  | Mean | Std. Deviation | N |
| Q81_1 Please read each statement and indicate how much the statement applied to you over the past week.  There are no right or wrong answers. Do not spend too much time on any one statement.-I found it hard to wind down | 1.39 | .965 | 976 |
| Q81_6 Please read each statement and indicate how much the statement applied to you over the past week.  There are no right or wrong answers. Do not spend too much time on any one statement.-I tended to over-react to situations. | .73 | .781 | 976 |
| Q81_8 Please read each statement and indicate how much the statement applied to you over the past week.  There are no right or wrong answers. Do not spend too much time on any one statement.-I felt I was using a lot of nervous energy. | .55 | .780 | 976 |
| Q81_11 Please read each statement and indicate how much the statement applied to you over the past week.  There are no right or wrong answers. Do not spend too much time on any one statement.-I found myself getting agitated | .63 | .789 | 976 |
| Q81_12 Please read each statement and indicate how much the statement applied to you over the past week.  There are no right or wrong answers. Do not spend too much time on any one statement.-I found it difficult to relax | .91 | .889 | 976 |
| Q81_14 Please read each statement and indicate how much the statement applied to you over the past week.  There are no right or wrong answers. Do not spend too much time on any one statement.-I was intolerant of anything that kept me from getting on with what I was doing | .71 | .845 | 976 |
| Q81_18 Please read each statement and indicate how much the statement applied to you over the past week.  There are no right or wrong answers. Do not spend too much time on any one statement.-I felt that I was rather touchy | .65 | .745 | 976 |

| **Summary Item Statistics** | | | | | | | |
| --- | --- | --- | --- | --- | --- | --- | --- |
|  | Mean | Minimum | Maximum | Range | Maximum / Minimum | Variance | N of Items |
| Item Means | .795 | .547 | 1.386 | .839 | 2.534 | .081 | 7 |
| Inter-Item Correlations | .525 | .388 | .663 | .275 | 1.708 | .007 | 7 |

| **Scale Statistics** | | | |
| --- | --- | --- | --- |
| Mean | Variance | Std. Deviation | N of Items |
| 5.57 | 19.852 | 4.456 | 7 |

### DASS Anxiety

| **Case Processing Summary** | | | |
| --- | --- | --- | --- |
|  | | N | % |
| Cases | Valid | 977 | 94.2 |
|  | Excluded^a^ | 60 | 5.8 |
|  | Total | 1037 | 100.0 |
| a. Listwise deletion based on all variables in the procedure. | | | |

| **Reliability Statistics** | | |
| --- | --- | --- |
| Cronbach's Alpha | Cronbach's Alpha Based on Standardized Items | N of Items |
| .824 | .848 | 7 |

| **Item Statistics** | | | |
| --- | --- | --- | --- |
|  | Mean | Std. Deviation | N |
| Q81_2 Please read each statement and indicate how much the statement applied to you over the past week.  There are no right or wrong answers. Do not spend too much time on any one statement.-I was aware of dryness of my mouth | .86 | 1.009 | 977 |
| Q81_4 Please read each statement and indicate how much the statement applied to you over the past week.  There are no right or wrong answers. Do not spend too much time on any one statement.-I experienced breathing difficulty (e.g. Excessively rapid breathing, breathlessness in the absence of physical exertion) | .22 | .559 | 977 |
| Q81_7 Please read each statement and indicate how much the statement applied to you over the past week.  There are no right or wrong answers. Do not spend too much time on any one statement.-I experienced trembling (e.g. in the hands) | .21 | .539 | 977 |
| Q81_9 Please read each statement and indicate how much the statement applied to you over the past week.  There are no right or wrong answers. Do not spend too much time on any one statement.-I was worried about situations in which I might panic and make a fool of myself. | .45 | .778 | 977 |
| Q81_15 Please read each statement and indicate how much the statement applied to you over the past week.  There are no right or wrong answers. Do not spend too much time on any one statement.-I felt I was close to panic | .28 | .629 | 977 |
| Q81_19 Please read each statement and indicate how much the statement applied to you over the past week.  There are no right or wrong answers. Do not spend too much time on any one statement.-I was aware of the action of my heart in the absence of physical exertion (e.g. heart rate increase, missing a beat) | .40 | .721 | 977 |
| Q81_20 Please read each statement and indicate how much the statement applied to you over the past week.  There are no right or wrong answers. Do not spend too much time on any one statement.-I felt scared without any good reason | .25 | .610 | 977 |

| **Summary Item Statistics** | | | | | | | |
| --- | --- | --- | --- | --- | --- | --- | --- |
|  | Mean | Minimum | Maximum | Range | Maximum / Minimum | Variance | N of Items |
| Item Means | .382 | .210 | .857 | .647 | 4.083 | .052 | 7 |
| Inter-Item Correlations | .444 | .259 | .691 | .432 | 2.672 | .014 | 7 |

| **Scale Statistics** | | | |
| --- | --- | --- | --- |
| Mean | Variance | Std. Deviation | N of Items |
| 2.68 | 11.978 | 3.461 | 7 |

### DASS Depression

| **Case Processing Summary** | | | |
| --- | --- | --- | --- |
|  | | N | % |
| Cases | Valid | 976 | 94.1 |
|  | Excluded^a^ | 61 | 5.9 |
|  | Total | 1037 | 100.0 |
| a. Listwise deletion based on all variables in the procedure. | | | |

| **Reliability Statistics** | | |
| --- | --- | --- |
| Cronbach's Alpha | Cronbach's Alpha Based on Standardized Items | N of Items |
| .912 | .914 | 7 |

| **Item Statistics** | | | |
| --- | --- | --- | --- |
|  | Mean | Std. Deviation | N |
| Q81_3 Please read each statement and indicate how much the statement applied to you over the past week.  There are no right or wrong answers. Do not spend too much time on any one statement.-I couldn’t seem to experience any positive feeling | .58 | .786 | 976 |
| Q81_5 Please read each statement and indicate how much the statement applied to you over the past week.  There are no right or wrong answers. Do not spend too much time on any one statement.-I found it difficult to work up the initiative to do things. | .71 | .808 | 976 |
| Q81_10 Please read each statement and indicate how much the statement applied to you over the past week.  There are no right or wrong answers. Do not spend too much time on any one statement.-I felt that I had nothing to look forward to | .38 | .742 | 976 |
| Q81_13 Please read each statement and indicate how much the statement applied to you over the past week.  There are no right or wrong answers. Do not spend too much time on any one statement.-I felt down-hearted and blue | .65 | .827 | 976 |
| Q81_16 Please read each statement and indicate how much the statement applied to you over the past week.  There are no right or wrong answers. Do not spend too much time on any one statement.-I was unable to become enthusiastic about anything | .44 | .726 | 976 |
| Q81_17 Please read each statement and indicate how much the statement applied to you over the past week.  There are no right or wrong answers. Do not spend too much time on any one statement.-I felt I wasn’t worth much as a person | .38 | .742 | 976 |
| Q81_21 Please read each statement and indicate how much the statement applied to you over the past week.  There are no right or wrong answers. Do not spend too much time on any one statement.-I felt that life was meaningless | .19 | .573 | 976 |

| **Summary Item Statistics** | | | | | | | |
| --- | --- | --- | --- | --- | --- | --- | --- |
|  | Mean | Minimum | Maximum | Range | Maximum / Minimum | Variance | N of Items |
| Item Means | .476 | .193 | .711 | .518 | 3.691 | .032 | 7 |
| Inter-Item Correlations | .602 | .424 | .747 | .323 | 1.762 | .007 | 7 |

| **Scale Statistics** | | | |
| --- | --- | --- | --- |
| Mean | Variance | Std. Deviation | N of Items |
| 3.33 | 17.889 | 4.230 | 7 |

## Reliability Burnout

### Personal burnout

| **Case Processing Summary** | | | |
| --- | --- | --- | --- |
|  | | N | % |
| Cases | Valid | 990 | 95.5 |
|  | Excluded^a^ | 47 | 4.5 |
|  | Total | 1037 | 100.0 |
| a. Listwise deletion based on all variables in the procedure. | | | |

| **Reliability Statistics** | | |
| --- | --- | --- |
| Cronbach's Alpha | Cronbach's Alpha Based on Standardized Items | N of Items |
| .904 | .907 | 6 |

| **Item Statistics** | | | |
| --- | --- | --- | --- |
|  | Mean | Std. Deviation | N |
| Q79_1 Please indicate how often you experience each of the following feelings: -How often do you feel tired? | 70.38 | 17.582 | 990 |
| Q79_2 Please indicate how often you experience each of the following feelings: -How often do you feel physically exhausted? | 59.47 | 20.000 | 990 |
| Q79_3 Please indicate how often you experience each of the following feelings: -How often do you feel emotionally exhausted? | 60.00 | 19.947 | 990 |
| Q79_4 Please indicate how often you experience each of the following feelings: -How often do you think “I can’t take this anymore?” | 42.73 | 25.688 | 990 |
| Q79_5 Please indicate how often you experience each of the following feelings: -How often do you feel worn out? | 56.64 | 22.303 | 990 |
| Q79_6 Please indicate how often you experience each of the following feelings: -How often do you feel weak and susceptible to burnout? | 46.16 | 25.172 | 990 |

| **Summary Item Statistics** | | | | | | | |
| --- | --- | --- | --- | --- | --- | --- | --- |
|  | Mean | Minimum | Maximum | Range | Maximum / Minimum | Variance | N of Items |
| Item Means | 55.896 | 42.727 | 70.379 | 27.652 | 1.647 | 101.619 | 6 |
| Inter-Item Correlations | .619 | .457 | .727 | .270 | 1.590 | .005 | 6 |

| **Scale Statistics** | | | |
| --- | --- | --- | --- |
| Mean | Variance | Std. Deviation | N of Items |
| 335.38 | 11739.366 | 108.348 | 6 |

### Burnout work

|  | | N | % |
| --- | --- | --- | --- |
| Cases | Valid | 978 | 94.3 |
|  | Excluded^a^ | 59 | 5.7 |
|  | Total | 1037 | 100.0 |
| a. Listwise deletion based on all variables in the procedure. | | | |

| **Reliability Statistics** | | |
| --- | --- | --- |
| Cronbach's Alpha | Cronbach's Alpha Based on Standardized Items | N of Items |
| .887 | .886 | 7 |

| **Item Statistics** | | | |
| --- | --- | --- | --- |
|  | Mean | Std. Deviation | N |
| RQ79_10 | 39.60 | 20.820 | 978 |
| Q79_7 Please indicate how often you experience each of the following feelings: -Do you feel worn out at the end of the working day? | 63.24 | 22.189 | 978 |
| Q79_8 Please indicate how often you experience each of the following feelings: -Are you exhausted in the morning at the thought of another day at work? | 44.12 | 24.832 | 978 |
| Q79_9 Please indicate how often you experience each of the following feelings: -Do you feel that every working hour is tiring for you? | 32.82 | 24.447 | 978 |
| Q80_1 For the following questions please indicate the intensity of your feelings-Is your work emotionally exhausting? | 52.04 | 25.637 | 978 |
| Q80_2 For the following questions please indicate the intensity of your feelings-Does your work frustrate you? | 44.35 | 26.516 | 978 |
| Q80_3 For the following questions please indicate the intensity of your feelings-Do you feel burnt out because of your work? | 36.68 | 28.943 | 978 |

| **Summary Item Statistics** | | | | | | | |
| --- | --- | --- | --- | --- | --- | --- | --- |
|  | Mean | Minimum | Maximum | Range | Maximum / Minimum | Variance | N of Items |
| Item Means | 44.694 | 32.822 | 63.241 | 30.419 | 1.927 | 104.935 | 7 |
| Inter-Item Correlations | .526 | .320 | .715 | .396 | 2.237 | .011 | 7 |

| **Item-Total Statistics** | | | | | |
| --- | --- | --- | --- | --- | --- |
|  | Scale Mean if Item Deleted | Scale Variance if Item Deleted | Corrected Item-Total Correlation | Squared Multiple Correlation | Cronbach's Alpha if Item Deleted |
| RQ79_10 | 273.26 | 15170.977 | .489 | .260 | .891 |
| Q79_7 Please indicate how often you experience each of the following feelings: -Do you feel worn out at the end of the working day? | 249.62 | 14138.159 | .660 | .458 | .874 |
| Q79_8 Please indicate how often you experience each of the following feelings: -Are you exhausted in the morning at the thought of another day at work? | 268.74 | 13129.238 | .768 | .623 | .860 |
| Q79_9 Please indicate how often you experience each of the following feelings: -Do you feel that every working hour is tiring for you? | 280.04 | 13369.574 | .734 | .586 | .864 |
| Q80_1 For the following questions please indicate the intensity of your feelings-Is your work emotionally exhausting? | 260.81 | 13489.026 | .666 | .470 | .873 |
| Q80_2 For the following questions please indicate the intensity of your feelings-Does your work frustrate you? | 268.51 | 13405.855 | .652 | .469 | .875 |
| Q80_3 For the following questions please indicate the intensity of your feelings-Do you feel burnt out because of your work? | 276.18 | 12206.907 | .793 | .637 | .856 |

| **Scale Statistics** | | | |
| --- | --- | --- | --- |
| Mean | Variance | Std. Deviation | N of Items |
| 312.86 | 18114.317 | 134.589 | 7 |

### Burnout Client

| **Case Processing Summary** | | | |
| --- | --- | --- | --- |
|  | | N | % |
| Cases | Valid | 989 | 95.4 |
|  | Excluded^a^ | 48 | 4.6 |
|  | Total | 1037 | 100.0 |
| a. Listwise deletion based on all variables in the procedure. | | | |

| **Reliability Statistics** | | |
| --- | --- | --- |
| Cronbach's Alpha | Cronbach's Alpha Based on Standardized Items | N of Items |
| .889 | .896 | 6 |

| **Item Statistics** | | | |
| --- | --- | --- | --- |
|  | Mean | Std. Deviation | N |
| Q79_14 Please indicate how often you experience each of the following feelings: -Do you find it hard to work with women? | 22.75 | 21.058 | 989 |
| Q79_15 Please indicate how often you experience each of the following feelings: -Does it drain your energy to work with women? | 27.73 | 21.668 | 989 |
| Q79_16 Please indicate how often you experience each of the following feelings: -Do you find it frustrating to work with women? | 25.86 | 20.692 | 989 |
| Q79_17 Please indicate how often you experience each of the following feelings: -Do you feel that you give more than you get back when you work with women? | 38.42 | 26.664 | 989 |
| Q79_18 Please indicate how often you experience each of the following feelings: -Are you tired of working with women? | 16.33 | 20.893 | 989 |
| Q79_19 Please indicate how often you experience each of the following feelings: -Do you sometimes wonder how long you will be able to continue working with women? | 22.47 | 25.796 | 989 |

| **Summary Item Statistics** | | | | | | | |
| --- | --- | --- | --- | --- | --- | --- | --- |
|  | Mean | Minimum | Maximum | Range | Maximum / Minimum | Variance | N of Items |
| Item Means | 25.594 | 16.330 | 38.423 | 22.093 | 2.353 | 54.574 | 6 |
| Inter-Item Correlations | .589 | .446 | .752 | .307 | 1.687 | .009 | 6 |

| **Item-Total Statistics** | | | | | |
| --- | --- | --- | --- | --- | --- |
|  | Scale Mean if Item Deleted | Scale Variance if Item Deleted | Corrected Item-Total Correlation | Squared Multiple Correlation | Cronbach's Alpha if Item Deleted |
| Q79_14 Please indicate how often you experience each of the following feelings: -Do you find it hard to work with women? | 130.81 | 8822.439 | .732 | .604 | .866 |
| Q79_15 Please indicate how often you experience each of the following feelings: -Does it drain your energy to work with women? | 125.83 | 8594.319 | .771 | .651 | .860 |
| Q79_16 Please indicate how often you experience each of the following feelings: -Do you find it frustrating to work with women? | 127.70 | 8720.536 | .780 | .664 | .859 |
| Q79_17 Please indicate how often you experience each of the following feelings: -Do you feel that you give more than you get back when you work with women? | 115.14 | 8610.962 | .574 | .342 | .895 |
| Q79_18 Please indicate how often you experience each of the following feelings: -Are you tired of working with women? | 137.23 | 8774.759 | .754 | .606 | .863 |
| Q79_19 Please indicate how often you experience each of the following feelings: -Do you sometimes wonder how long you will be able to continue working with women? | 131.09 | 8282.055 | .685 | .531 | .874 |

| **Scale Statistics** | | | |
| --- | --- | --- | --- |
| Mean | Variance | Std. Deviation | N of Items |
| 153.56 | 12162.764 | 110.285 | 6 |

# Association between burnout and DASS subscales

## Spearman correlations between Burnout and DASS

(nonparametric tests used due to skewed DASS scores)

| **Correlations** | | | | | |
| --- | --- | --- | --- | --- | --- |
|  | | | DASSStress | DASSAnx | DASSDep |
| Spearman's rho | TBurn_Personal | Correlation Coefficient | .592^**^ | .508^**^ | .619^**^ |
|  |  | Sig. (2-tailed) | .000 | .000 | .000 |
|  |  | N | 975 | 976 | 975 |
|  | TBurn_WorkCorrect | Correlation Coefficient | .632^**^ | .533^**^ | .633^**^ |
|  |  | Sig. (2-tailed) | .000 | .000 | .000 |
|  |  | N | 968 | 969 | 968 |
|  | TBurn_ClientCorrect | Correlation Coefficient | .398^**^ | .313^**^ | .392^**^ |
|  |  | Sig. (2-tailed) | .000 | .000 | .000 |
|  |  | N | 974 | 975 | 974 |
| **. Correlation is significant at the 0.01 level (2-tailed). | | | | | |

## Boxplots of Burnout-Work with DASS in 2 groups

### Anxiety


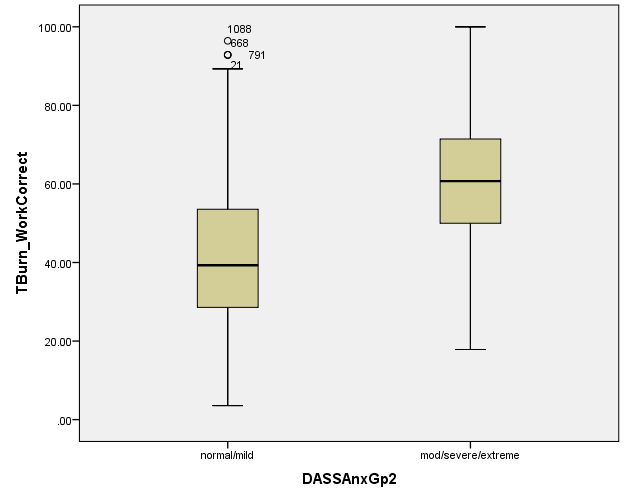


### Depression


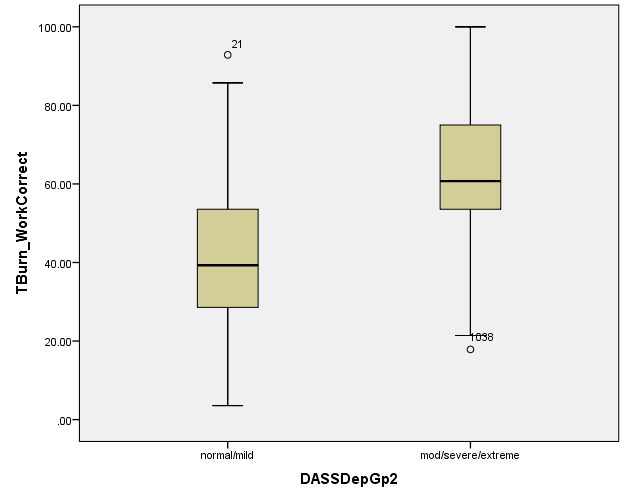


### Stress


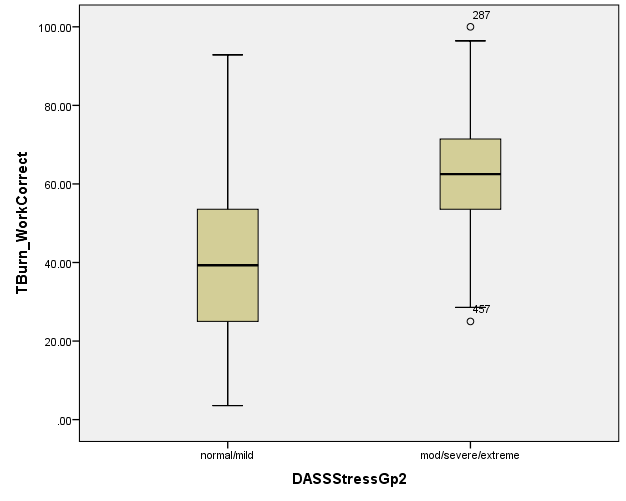


## Compare Burnout scores across DASS clinical 2 groups

### Stress

**Mann-Whitney Test**

| **Ranks** | | | | |
| --- | --- | --- | --- | --- |
|  | DASSStressGp2 | N | Mean Rank | Sum of Ranks |
| TBurn_WorkCorrect | 1 normal/mild | 754 | 412.50 | 311023.50 |
|  | 2 mod/severe/extreme | 214 | 738.19 | 157972.50 |
|  | Total | 968 |  |  |

| **Test Statistics^a^** | |
| --- | --- |
|  | TBurn_WorkCorrect |
| Mann-Whitney U | 26388.500 |
| Wilcoxon W | 311023.500 |
| Z | -15.062 |
| Asymp. Sig. (2-tailed) | .000 |
| a. Grouping Variable: DASSStressGp2 | |

| **Report** | | |
| --- | --- | --- |
| TBurn_WorkCorrect | | |
| DASSStressGp2 | N | Median |
| 1 normal/mild | 754 | 39.2857 |
| 2 mod/severe/extreme | 214 | 62.5000 |
| Total | 968 | 42.8571 |

### Depression

**Mann-Whitney Test**

| **Ranks** | | | | |
| --- | --- | --- | --- | --- |
|  | DASSDepGp2 | N | Mean Rank | Sum of Ranks |
| TBurn_WorkCorrect | 1 normal/mild | 800 | 429.60 | 343681.00 |
|  | 2 mod/severe/extreme | 168 | 745.92 | 125315.00 |
|  | Total | 968 |  |  |

| **Test Statistics^a^** | |
| --- | --- |
|  | TBurn_WorkCorrect |
| Mann-Whitney U | 23281.000 |
| Wilcoxon W | 343681.000 |
| Z | -13.351 |
| Asymp. Sig. (2-tailed) | .000 |
| a. Grouping Variable: DASSDepGp2 | |

| **Report** | | |
| --- | --- | --- |
| TBurn_WorkCorrect | | |
| DASSDepGp2 | N | Median |
| 1 normal/mild | 800 | 39.2857 |
| 2 mod/severe/extreme | 168 | 60.7143 |
| Total | 968 | 42.8571 |

### Anxiety

**Mann-Whitney Test**

| **Ranks** | | | | |
| --- | --- | --- | --- | --- |
|  | DASSAnxGp2 | N | Mean Rank | Sum of Ranks |
| TBurn_WorkCorrect | 1 normal/mild | 771 | 427.00 | 329217.00 |
|  | 2 mod/severe/extreme | 198 | 710.85 | 140748.00 |
|  | Total | 969 |  |  |

| **Test Statistics^a^** | |
| --- | --- |
|  | TBurn_WorkCorrect |
| Mann-Whitney U | 31611.000 |
| Wilcoxon W | 329217.000 |
| Z | -12.749 |
| Asymp. Sig. (2-tailed) | .000 |
| a. Grouping Variable: DASSAnxGp2 | |

| **Report** | | |
| --- | --- | --- |
| TBurn_WorkCorrect | | |
| DASSAnxGp2 | N | Median |
| 1 normal/mild | 771 | 39.2857 |
| 2 mod/severe/extreme | 198 | 60.7143 |
| Total | 969 | 42.8571 |
